# Novel ventilation techniques in children

**DOI:** 10.1111/pan.14344

**Published:** 2021-12-05

**Authors:** André Dos Santos Rocha, Walid Habre, Gergely Albu

**Affiliations:** ^1^ Division of Anesthesiology and Unit for Anesthesiological Investigations Department of Acute Medicine University Hospitals of Geneva and University of Geneva Geneva Switzerland; ^2^ Pediatric Anesthesia Unit Department of Acute Medicine University Hospitals of Geneva Geneva Switzerland

**Keywords:** flow‐controlled ventilation, negative pressure ventilation, pressure support ventilation, pressure‐regulated volume control, variable ventilation, ventilator‐induced lung injury

## Abstract

Extraordinary progress has been made during the past few decades in the development of anesthesia machines and ventilation techniques. With unprecedented precision and performance, modern machines for pediatric anesthesia can deliver appropriate mechanical ventilation for children and infants of all sizes and with ongoing respiratory diseases, ensuring very small volume delivery and compensating for circuit compliance. Along with highly accurate monitoring of the delivered ventilation, modern ventilators for pediatric anesthesia also have a broad choice of ventilation modalities, including synchronized and assisted ventilation modes, which were initially conceived for ventilation weaning in the intensive care setting. Despite these technical advances, there is still room for improvement in pediatric mechanical ventilation. There is a growing effort to minimize the harm of intraoperative mechanical ventilation of children by adopting the protective ventilation strategies that were previously employed only for prolonged mechanical ventilation. More than ever, the pediatric anesthesiologist should now recognize that positive‐pressure ventilation is potentially a harmful procedure, even in healthy children, as it can contribute to both ventilator‐induced lung injury and ventilator‐induced diaphragmatic dysfunction. Therefore, careful choice of the ventilation modality and its parameters is of paramount importance to optimize gas exchange and to protect the lungs from injury during general anesthesia. The present report reviews the novel ventilation techniques used for children, discussing the advantages and pitfalls of the ventilation modalities available in modern anesthesia machines, as well as innovative ventilation modes currently under development or research. Several innovative strategies and devices are discussed. These novel modalities are likely to become part of the armamentarium of the pediatric anesthesiologist in the near future and are particularly relevant for challenging ventilation scenarios.

## 1 INTRODUCTION

Mechanical ventilation is required for many procedures in which a child undergoes general anesthesia. Remarkable technical advances have been achieved for pediatric anesthesia machines, with improved performance in terms of accuracy, volume delivery, and choice of ventilation modalities, which is currently equivalent to those offered by pediatric intensive care ventilators. Despite these technical improvements, mechanical ventilation has been increasingly recognized as a potentially harmful procedure in itself, especially for pediatric patients with underlying or ongoing lung injury.^1^, ^2^

The harm generated by ventilation, commonly termed ventilator‐induced lung injury (VILI), is usually prevented by strategies of lung‐protective ventilation. These protective strategies combine pressure, volume, and oxygen thresholds which were shown to limit the injury of mechanical ventilation and even reduce the mortality in adults.^3^ However, as of today, there are still no pediatric counterparts to the adult ARDS Network trial, nor has there been any evidence‐based approach on the definition or guidelines of pediatric protective ventilation. Thus, adult practices for protective ventilation are often adopted to guide pediatric ventilation, even for children with healthy lungs.

It must be stressed that children experience important dynamic changes in their respiratory systems that take place from birth until adolescence, including lung growth and development, alveolar multiplication and chest‐wall modifications due to ossification and increased musculature. These aspects explain why “children are not little adults,” a common catchphrase used to explain why adult recommendations might not fit the pediatric population. Since there is little evidence to guide ventilation strategies for children—with or without lung injury—studies are required to assess the effects of different ventilation modes.

**I**t remains unclear whether the use of different ventilation modes or certain respiration techniques (eg, apneic oxygenation, high‐flow nasal oxygenation) results in any meaningful clinical benefit for the pediatric surgical patient. Comparative studies between ventilation modes used with children are scarce and most use surrogate outcome markers to determine the effectiveness of ventilation modalities. In fact, it is difficult to demonstrate differences in clinical outcomes of anesthetized, ventilated children given that most surgical patients have healthy lungs and would not potentially benefit from an improved ventilation strategy.

In addition, data on the mechanisms and relevance of VILI in the pediatric population are highly debatable. Hence, most of the practice and recommendations in pediatric ventilation stem from experience and extrapolation from adult research.

## 2 NEW VENTILATION MODES IN PEDIATRIC ANESTHESIA

The “new” ventilation modes in use during intraoperative ventilation for children are, in fact, modes which were initially used in the intensive care unit (ICU) setting, either for weaning patients from ventilatory support or for optimizing the pressure‐volume curve during prolonged ventilation. The recognition that VILI and ventilator‐induced diaphragm dysfunction can happen to individuals under mechanical ventilation,^1^, ^4^ prompted anesthesiologists to adopt new intraoperative ventilation strategies that are meant to reduce VILI in all patients who receive positive‐pressure ventilation, including those without preoperative lung disorders.

Although data are scarce for the relevance of VILI in the pediatric population, the new ventilation modes used in the intraoperative period take into consideration the principles of lung‐protective ventilation, along with an attempt to optimize the patient‐ventilator synchrony, work of breathing, and respiratory mechanics.

In this section, we discuss the benefits and pitfalls of the “new” ventilation modes in the intraoperative setting. Our focus will be on pressure support ventilation during spontaneous breathing and on the pressure‐regulated volume mode during mandatory ventilation.

## 3 PRESSURE SUPPORT VENTILATION

Modern anesthesia ventilators have the ability to provide pressure support ventilation (PSV) to assist spontaneous breathing for the intubated child. Indeed, the effects of general anesthesia and the ventilation circuit impose increased work of breathing. PSV can facilitate the work of breathing by delivering inspiratory pressure and allowing the child to determine the inspiratory time. The use of positive end‐expiratory pressure (PEEP) should be routinely applied to prevent atelectasis^5^ and to optimize the respiratory mechanics in anesthetized children with healthy lungs.^6^ The use of inspiratory pressure support along with PEEP can decrease the work of breathing, improve gas exchange and be considered for children regardless of age.

In the absence of PSV, the maintenance of spontaneous breathing during anesthesia procedures may have deleterious consequences. Anesthesia leads to dose‐dependent hypotonia of superior airways,^7^, ^8^ atelectasis formation with decreased functional residual capacity and reduced tidal volume and minute ventilation.^9^ These consequences can be counteracted with PSV, by decreasing the work of breathing and restoring the minute ventilation for children with endotracheal tubes and supraglottic devices.^10^ For adults, it has been demonstrated that PSV reduces the work of breathing and improves the quality of preoxygenation and the delivery of halogenated anesthetics.^11^, ^12^, ^13^ In addition, PSV during general anesthesia is also capable of reducing intraoperative and postoperative atelectasis.^14^, ^15^ The specificities of respiratory anatomy and physiology in children make the advantages of PSV particularly relevant, especially considering the shorter delay to hemoglobin oxygen desaturation and the high rate of perioperative respiratory complications (PRCs).^16^, ^17^

It is likely that PSV is currently underused for anesthesia procedures under spontaneous breathing. Data collected in 2014 and 2015, including 31 024 anesthetic procedures in 261 hospitals in Europe, demonstrated that unassisted ventilation is more often chosen than PSV during spontaneous breathing.^18^ For children with airways secured with a tracheal tube, the use of PSV was reported in 6.3% of the procedures compared to 6.4% with unassisted spontaneous breathing. However, with supraglottic airway devices, unassisted spontaneous breathing was chosen three times more often than PSV (45.5% versus 12.9%).

The misuse or misunderstanding of PSV can also generate deleterious effects. Unlike with mandatory modes, issues such as the respiratory drive, synchrony, and ventilator‐valve opening settings should be considered when applying this ventilation strategy. While applying PSV, the child must have a respiratory drive compatible with assisted ventilation. The trigger settings must be decreased for small children, usually for values <1 L/min, but auto‐triggering must be avoided. Subsequently, the clinician must set a backup ventilation mode with an appropriate rate and pressure in case the respiratory drive of the child is suppressed during the anesthesia procedure. Another aspect that requires careful assessment by the anesthetist is the child‐ventilator synchrony. In addition to regular adjustment of the level of pressure support to ensure optimized work of breathing, the clinician may need to change the expiratory valve opening settings (cycling‐off flow threshold) and the pressurization time, taking the respiratory condition into consideration. If these aspects are neglected, premature or delayed cycling, dynamic hyperinflation, increased work of breathing or even barotrauma can occur. Typically, the cyclic‐off, which is often set at 25% of the inspiratory flow, should be decreased in the presence of restrictive disease and increased in the event of an obstructive condition.

Considering all of the above, it is no surprise that the use of PSV requires an appropriate setting and possibly closer monitoring than unassisted or mandatory ventilation. However, there are multiple benefits of using PSV during general anesthesia. In comparison with pressure‐controlled ventilation (PCV), PSV significantly decreases the propofol consumption and the emergence time and improves the oxygenation index for children.^19^ Likewise, PSV was shown to improve the gas exchange in comparison with continuous positive airway pressure (CPAP).^10^ The maintenance (and support) of spontaneous ventilation in surgical patients has many clinical advantages, including the titration of analgesia and depth of anesthesia, the potential of improving hemodynamics, and the facilitation of emergence. Therefore, although the European data revealed that PSV is not often applied, we encourage its application whenever spontaneous breathing is maintained throughout general anesthesia procedures.

## 4 PRESSURE‐REGULATED VOLUME CONTROL

Mandatory modes of mechanical ventilation are designed to deliver controlled ventilation (preset volume or pressure) while the child makes no respiratory effort under general anesthesia. In pediatric anesthesia, volume‐controlled ventilation (VCV) is particularly challenging because the target volume can be dangerously dependent on respiratory circuit compliance. The use of PCV overcomes many typical limitations of anesthesia machines and the respiratory circuit; however, the primary disadvantage of PCV is the lack of a guaranteed volume. Both PCV and VCV deliver the settings preselected by the clinician and will not accommodate changes in respiratory compliance, which is a parameter that changes rapidly during surgical procedures (eg, laparoscopy, surgical maneuvers).

An alternative ventilation mode can assess the respiratory compliance on a breath‐by‐breath basis and adapt the pressure level required to deliver a set volume target. The result is a PCV‐like decelerating flow delivery with a nearly constant tidal volume, similar to VCV. This mode is referred to by various terms from different manufacturers: pressure‐regulated volume control (PRVC), PCV‐volume guarantee (PCV‐VG), pressure‐regulated volume‐target ventilation (PRVT), etc. By guaranteeing the tidal volume, anesthesiologists can avoid sudden changes in carbon dioxide, which may have deleterious effects on both cerebral and pulmonary circulation.

PRVC incorporates the benefits of VCV and PCV in a single ventilation mode. Despite this advantage, PRVC is rarely used in the pediatric anesthesia environment, as reported by the APRICOT epidemiological data.^18^ PRVC was used in less than 7% of the procedures for children with an endotracheal tube and less than 3% with a supraglottic device. In neonates, who are more prone to sudden changes in chest and lung compliance, only 3.5% of the procedures were performed with PRVC (72% were ventilated with PCV and 17% with VCV).

In some settings, PRVC can indeed have advantages over VCV, as demonstrated for infants after cardiac surgery, in which a significant decrease in peak inspiratory pressure was achieved with PRVC without affecting gas exchange in contrast to VCV.^20^ In other settings, however, VCV may be superior to PRVC, particularly for children with impaired respiratory mechanics due to severe asthma. The application of PRVC in severely obstructed patients often results in hypoventilation,^21^, ^22^ since the ventilator fails to deliver the programmed tidal volume with the decelerating flow mode in the presence of high‐resistance lung diseases, preventing the use of low I:E ratios, which are needed to avoid air trapping.

In summary, PRVC can optimize the pressure‐volume curve in most situations by ensuring the set tidal volume at the lowest inspiratory pressure, adapting the delivered pressure to the dynamic compliance of the respiratory system. Thus, this mode is suitable to most surgical pediatric procedures for which children without preexisting lung disorders receive general anesthesia. Caution should be applied if PRVC is used to ventilate children with injured and obstructed lungs.

## 5 NOVEL VENTILATION STRATEGIES AND DEVICES

Despite great advances in the technical aspects of modern anesthesia machines and in the comprehension of VILI and patient‐ventilator interaction, we still witness a considerable amount of PRCs, especially after lengthy general anesthesia procedures. Thus, there is a clear need to further develop new ventilation strategies (and devices) that ensure adequate gas exchange while reducing the shear stress and strain that can lead to VILI.

Some experimental and clinical approaches have been reported in the recent literature, including new ventilation concepts and devices that may be considered for intraoperative mechanical ventilation. In this section, we summarize novel ventilation techniques which may become available for challenging ventilation scenarios or which have the potential of preventing VILI and/or improving gas exchange inside and outside the operating room.

## 6 THE VENTRAIN^®^ SYSTEM

Technical progress in mechanical ventilation has been spectacular in recent years, resulting in new ventilation strategies and sophisticated ventilators with new ventilation modes. Difficult situations, however, sometimes require simple solutions. Such situations include difficult airways or “cannot intubate, cannot oxygenate” (CICO) scenarios. The algorithm of the Difficult Airway Society has not been changed since 2015, although new technologies and devices have been introduced to the market. It is worth being familiar with some of these innovations, as they may become valuable tools in our armamentarium for CICO scenarios.

**FIGURE 1:**
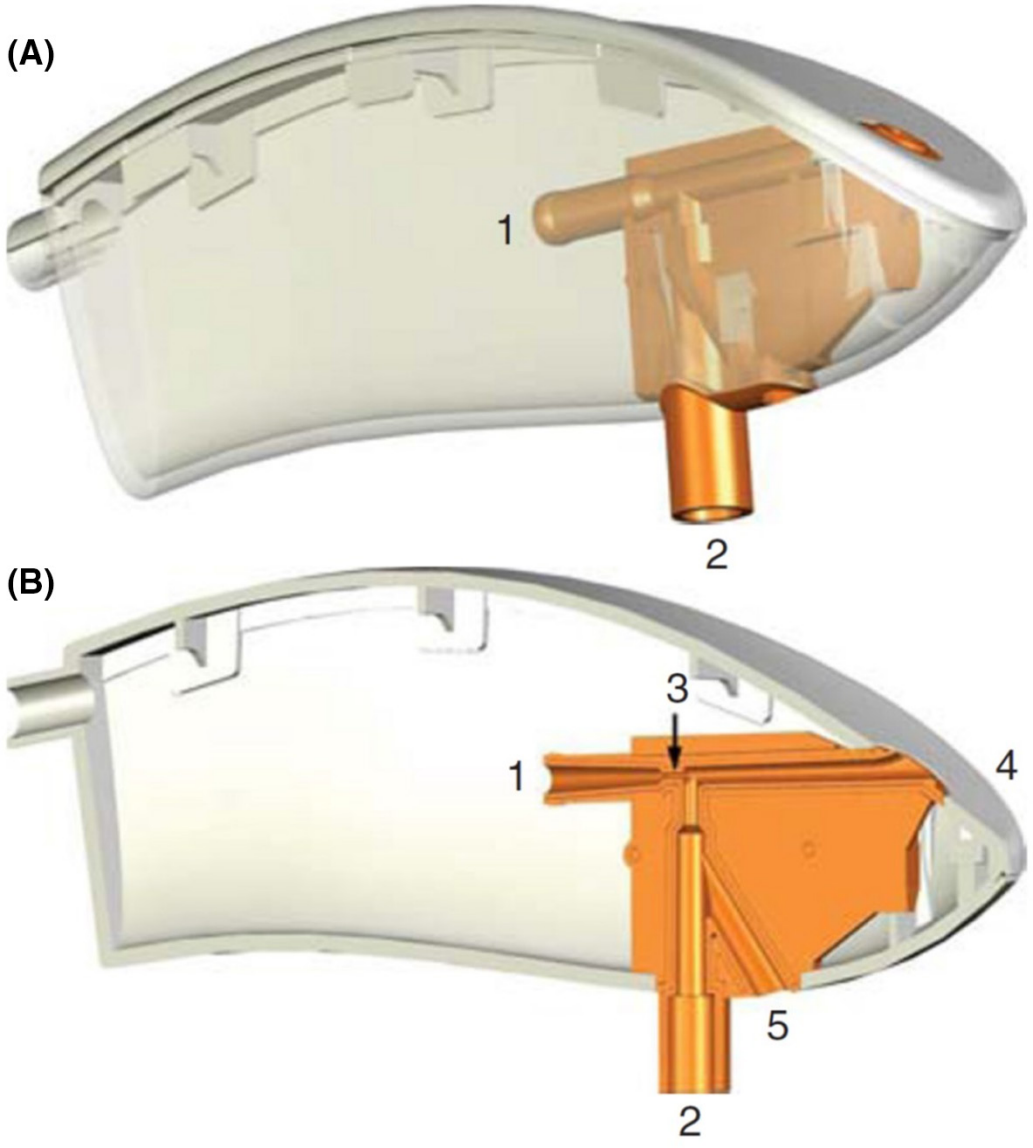
(A) Schematic model of the Ventrain^®^ device. The shell is shown transparently, with the oxygen inlet (1) and the outflow port toward the patient (2). (B) Cross‐section illustration of the Ventrain^®^ showing the oxygen inlet (1), the outflow port toward the patient (2), a narrowing part to increase the gas velocity (3) toward the outflow port (4) which can be manually occluded for inspiration and released for expiration; an outflow port for pressure equilibration (5) can be occluded by the user during ventilation and released for pressure equilibration. Reproduced with permission from^47^

The Ventrain^®^ device (Figure 1) is a manually operated, portable ventilation system which connects a pressurized oxygen source (an oxygen bottle or a standard wall outlet with a flow regulator) to many different airway cannulas or catheters with a Luer‐lock connection. The device is basically a T‐piece with gas inflow on one side and two outflow ports on the other. One outflow can be manually occluded by the user, directing the flow toward the patient for inspiration; this port must be released for expiration. During expiration, a narrowing in the inner tubing before the patient outlet accelerates the flow and exerts a suction effect on the airflow coming from the patient (active expiration) due to the Venturi effect.

The Ventrain^®^ device provides the noticeable advantage that its operation does not require high pressures but requires only a stable flow, as opposed to, for example, jet ventilation. The shorter (and active) expiratory time permits higher minute volume and prevents pressure build up, barotrauma and air trapping with consequent deleterious hemodynamic effects. Another main advantage is expiratory ventilation assistance capable of counteracting the expiratory flow limitation that arises from narrow tracheal tubes. Thus, the Ventrain^®^ device facilitates both oxygenation and CO_2_ removal when ventilating through small lumen tubes. Overall, this device has the promise to become an alternative for pediatric airway emergencies (eg, anaphylaxis and upper airway infection). Accordingly, the use of Ventrain^®^ in pediatric anesthesia has been limited to emergency situations, with only a few cases reported in the literature so far. A case of failed intubation of a 2.1 kg premature baby with glottis edema, severe desaturation (SpO_2_ <40%), and severe bradycardia (40 bpm) was managed with an 8 Fr Frova catheter and then successfully ventilated with the Ventrain^®^ system, resolving the vital problem.^23^ A second case was reported for a failed intubation of a 4.3 kg baby that could be rescued with the Ventrain^®^ device connected to a Cook tube exchange catheter (ID 1.66 mm). After stabilization, a 3 mm ID endotracheal tube was used to secure the airway with an over‐the‐catheter technique and the help of video laryngoscopy.^23^ Other life‐saving uses have been reported, such as the use of Ventrain^®^ associated with rigid bronchoscopy to stabilize a 3.7 kg baby with respiratory distress after laryngeal microsurgery^24^ and as salvage therapy when the PICU ventilator failed to ventilate due to extremely high peak pressures.^24^

One disadvantage of the Ventrain^®^ system is its requirement for manual operation. Considering that pediatric CICO situations are highly stressful, the clinician must be able to operate the flow ports of the Ventrain^®^ device while facing a life‐threatening situation. Another disadvantage is the lack of continuous pressure monitoring, making observation of chest movements crucial for minimizing the risk of lung injury.

## 7 FLOW‐CONTROLLED VENTILATION

**FIGURE 2:**
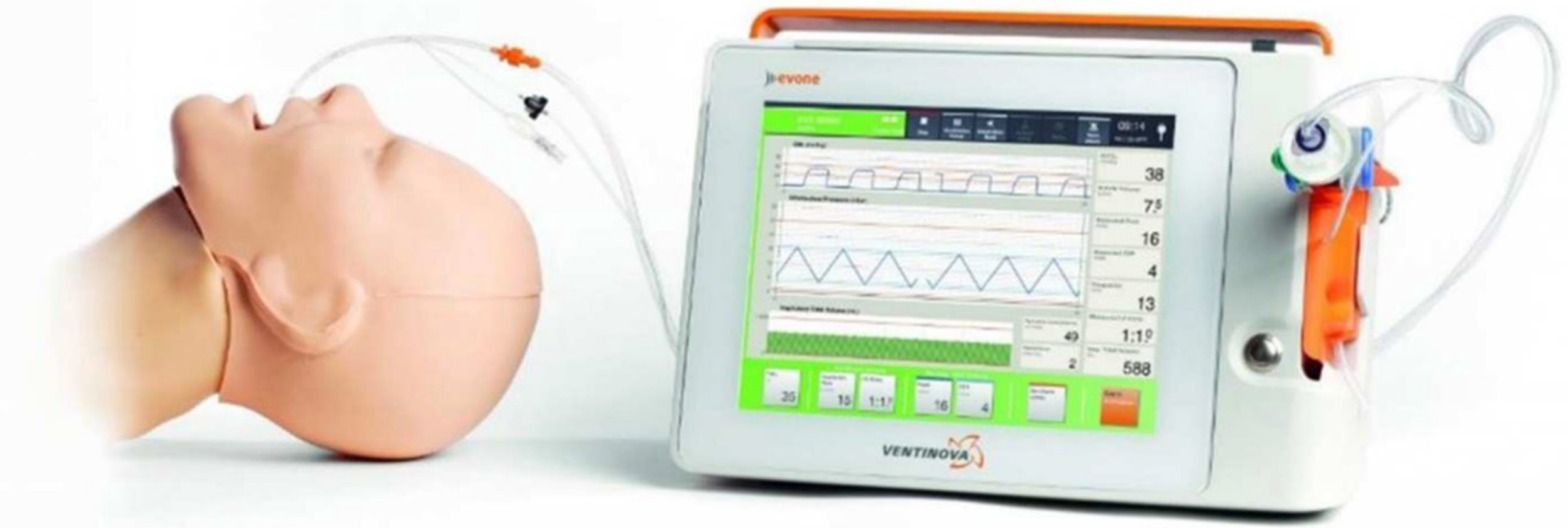
Evone ventilator (Ventinova Medical B.V., Eindhoven, the Netherlands) connected to a mannequin with the Tritube for flow‐controlled ventilation. Reproduced with permission from^48^

The energy dissipation that occurs during intermittent positive‐pressure ventilation is one of the contributors to VILI. In 2018, a mathematical model was used to demonstrate that the element responsible for the dissipated energy throughout the ventilation cycle is the varying flow.^25^ Therefore, to reduce energy dissipation, the flow must be “controlled,” that is, held constant with an inspiratory to expiratory ratio (I:E) close to 1:1. This control has become possible with the development of the Evone^®^ flow‐controlled ventilator (FCV, Figure 2). This ventilator uses technology based on the Bernoulli principle, with an active expiratory phase using the Venturi effect which allows expiratory flow limitations to be overcome and ventilation through very narrow internal diameter endotracheal tubes, such as the Tritube^®^. The Tritube^®^ comes with an internal diameter smaller than 3 mm but with an inflatable cuff to secure the airway. This technology creates linear changes in inspiratory and expiratory intratracheal pressure with very low hysteresis. As flow can be kept constant and stable both during inspiration and expiration with a I:E ratio of 1:1, FCV can reduce the applied mechanical energy and, therefore, energy dissipation.

In addition, the use of relatively low flow rates with FCV leads to improved distribution of lung aeration and improved gas exchange compared to conventional ventilation. Experimental data in models of acute respiratory distress syndrome (ARDS) demonstrated that FCV can improve oxygenation, increase the normally ventilated lung area, and decrease lung tissue inflammation.^26^

A remarkable advantage of this technology is the possibility to ventilate through very narrow endotracheal tubes. This can be advantageous for upper airway surgery and in difficult intubation scenarios. A randomized controlled trial of laryngeal surgery for adults that compared a microlaryngeal tube of 6.0 mm with VCV and the Tritube^®^ with FCV demonstrated that the latter strategy improved glottic visibility and lung aeration measured by electrical impedance tomography.^27^

Despite the aforementioned advantages of FCV, including the lung‐protective ventilation, it also has some limitations. In its current setting, Evone® FCV can only be used with total intravenous anesthesia since vaporizers cannot be included in the breathing circuit. This aspect is highly relevant in pediatric anesthesia. The system lacks adaptive ventilation modes and the possibility to be triggered by the patient; hence, patient‐ventilator synchrony or weaning can be problematic. Although there is some exciting evidence for better gas exchange and protective ventilation, there is a lack of hard evidence for its use in different situations (eg, pediatric anesthesia) or pathological conditions (eg, asthma). At present, the Evone^®^ FCV is only recommended by the manufacturer for use with an ideal body weight of over 40 kg. Further research is required to clarify whether there are advantages from FCV for the pediatric population. Furthermore, the Evone^®^ ventilator might also require technical adjustments to operate with the low tidal volumes necessary to ensure safe ventilation for children.

## 8 PHYSIOLOGICALLY VARIABLE VENTILATION

A mandatory ventilation mode that mimics spontaneous breathing by incorporating breath‐by‐breath variations in tidal volume and respiratory rate, termed physiologically variable ventilation (PVV), has been proposed as less injurious than conventional ventilation and capable of improving gas exchange.

The rationale for PVV is based on the fact that variability, or “noise,” defines the pace of several organic phenomena, including healthy breathing. These natural breathing variations are beneficial for lung structure and function.^28^ PVV can lead to recruitment and stabilization of airspaces, contributing to gas exchange, as well as to an improvement in ventilation/perfusion (V/Q) matching.^29^ The variable ventilation pattern has the ability to optimize the time constant in the heterogenous airspaces across the lung and, by doing so, to increase the surface area for gas exchange. In addition, a perfusion redistribution from dependent to non‐dependent lung zones has also been documented.^30^ Finally, microstructural effects also play a role, since the variable stretch in the respiratory epithelium has been shown to increase alveolar stability, surfactant production, and ameliorate inflammation.^31^

This ventilation mode has been mostly studied in experimental settings using models of healthy and injured lungs, including ARDS, asthma, and chronic obstructive pulmonary disease.^32^, ^33^, ^34^, ^35^ Summing up the various experimental scenarios, PVV consistently prevented the deterioration of respiratory tissue mechanics, ventilatory pressures, oxygenation, intrapulmonary shunt fraction, and inflammation that was observed over time with conventional (monotonous) ventilation modes.

Clinical data using PVV are not available in the pediatric field; however, a clinical trial comparing conventional VCV with variable ventilation with adults undergoing general anesthesia for abdominal aortic surgery has reported improved lung function with the variable mode, including improvements in arterial oxygen and carbon dioxide partial pressure, lower dead space ventilation, increased compliance, and reduced inspiratory pressure.^36^

Interestingly, there is evidence that variable ventilation can improve lung function during both mandatory and assisted modes,^37^ demonstrating that variable pressure support along with the patient's breathing effort does not compromise synchrony.

Despite a considerable number of promising reports published since the proposal of a “computer‐controlled mechanical ventilation programmed for biological variability” 25 years ago,^34^ a variable ventilation mode is still not commercially available for clinical use.

## 9 NEGATIVE PRESSURE VENTILATION

The use of negative pressure ventilation (NPV), which was extensively used in the form of the iron lung, almost completely disappeared after the poliomyelitis outbreaks in the late 1950s. The transition to positive‐pressure ventilation started in 1952 with a 12‐year‐old girl, Vivi Ebert, whose respiratory failure caused by polio failed to be treated with the iron lung. Urgent tracheostomy and positive‐pressure ventilation saved her life and she lived 20 years after the intervention.^38^ With the advent of positive‐pressure ventilators, which were considerably smaller, more performant and less cumbersome and risky than the iron lung, the use of NPV gradually disappeared. Positive‐pressure ventilation with secured airways became the standard method for mechanical ventilation; however, shortly after its widespread acceptance, cases with structural lung damage were documented and the phenomena were later referred to as VILI. Different ventilation strategies have been established to reduce the incidence of VILI, but in some cases (eg, ARDS) ventilation can be difficult and injurious. Technical progress provided different alternatives to reduce VILI, including new tools in the field of noninvasive ventilation (NIV). These new alternatives became particularly relevant during the COVID‐19 pandemic, in which our paradigm changed from invasive ventilation toward different options of NIV, such as CPAP machines and high‐flow nasal oxygenation. In many cases, NIV could be used to avoid intubation, muscle paralysis, and sedation. Currently, NIV is used as a prophylactic or therapeutic tool and in different bridging and weaning strategies.

In modern times, NPV has been reinvented through the noninvasive cuirass ventilators. These ventilators consist of a plastic shell connected to a ventilator that can be strapped around the chest to generate negative pressure. The design allows a considerably larger degree of freedom for the patient (and the caretaker) than its predecessor, the iron lung, allowing the patient to use their hands, talk, eat, and drink.

**FIGURE 3:**
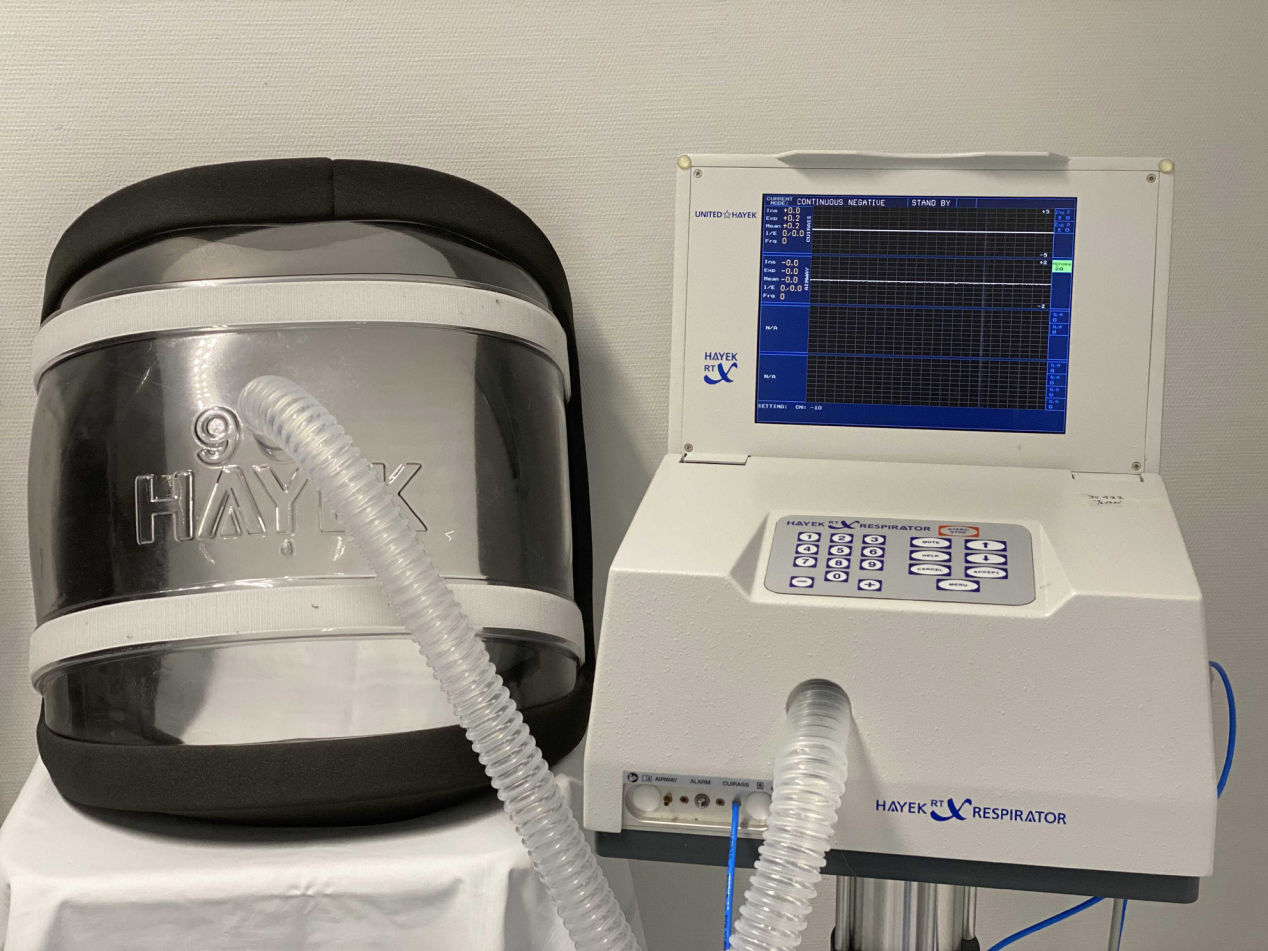
Hayek RTX Biphasic Cuirass Ventilator. The ventilator is connected to the cuirass shell with a tubing. The flexible cuirass shell is available in different sizes ranging from small neonatal size to large adult size

The biphasic cuirass ventilation (BCV, Figure 3) device can be used in different modes: It can be used in a continuous negative pressure or a biphasic mode, suitable for controlled or triggered ventilation (even with cardiac synchronization), or can deliver a high‐frequency oscillation mode that can help with physiotherapy and to clear secretions.

There are numerous studies published on the use of cuirass ventilators in pediatrics, but most of these are case reports or with a small number of participants. Hassinger and colleagues^39^ published a study on the use of NPV in pediatric acute respiratory failure that involved 233 children. Although it is a retrospective study on a heterogeneous population, they demonstrated a decline of the annual percentage of pediatric ICU admissions requiring intubation by 28% in the 3‐year period following the introduction of NPV to their institution. The median age of the patients was 15.5 months; 70% of the patients were considered responders to NPV with a very low complication rate of only 3% (hypothermia, skin lesions, and gastroesophageal reflux). It is important to mention that, in this study, NPV was not used as first line therapy and NPV settings, initiation and titration were at the discretion of the managing medical team because no protocol was in place.

Based on our knowledge on the differences between positive and NPV, cardiac patients with hemodynamic consequences could be potential candidates for NPV. Shime and colleagues^40^ studied the effect of the continuous negative extrathoracic pressure (CNEP) mode of the cuirass ventilator on children after surgery for congenital heart defects. NPV was used after extubation for small children (aged 1–34 months) who underwent different types of heart surgery. They demonstrated improved arterial oxygenation, decreased superior vena cava pressure, and increased urinary output as signs of improved hemodynamics under CNEP use. Since this study lacks a control group, conclusions should be considered with caution since postoperative improvement could be the natural course of these patients and CNEP was used only prophylactically. Shekerdemian and colleagues used NPV in the acute postoperative period after tetralogy of Fallot repair and studied the temporal influences on hemodynamic changes.^41^ NPV was used as a complementary method for intermittent positive‐pressure ventilation for intubated patients. This complex study involved 23 patients with different durations of NPV application and different subgroup analyses (patients with restrictive right ventricular physiology *versus* nonrestrictive patients); overall, they demonstrated a significant improvement in the cardiac output under NPV.

The use of NPV with clinical benefits was also reported for numerous noncardiac pathological conditions that could result in respiratory failure. A single‐center study reported the use of NPV for acute respiratory failure support in 118 children with bronchiolitis and pneumonia.^42^ NPV was also successfully used in neuromuscular disorders such as congenital myotonic dystrophy^43^ and in a case in which tension pneumothorax developed under noninvasive positive‐pressure ventilation in a boy with nemaline myopathy.^44^ Stone and colleagues^45^ used BCV for children with asthma and bronchiolitis to avoid intubation and demonstrated better oxygenation and decreased work of breathing as clinical benefit. Finally, Mori and colleagues successfully treated with BCV a 7‐year‐old patient with Swyer‐James syndrome and respiratory distress complicated by atelectasis.^46^

Overall, cuirass ventilators with different modes of NPV have the potential to be used with important clinical benefit for a wide variety of pathological conditions. It conferred benefits to cardiac patients by improving hemodynamics, in weaning from positive‐pressure ventilation or combined with it, and finally as a bridge therapy until intubation or lung transplantation became available. BCV was also used to avoid intubation in selected patients. It could potentially be involved in physiotherapy, home care, or even palliative care with a better quality of end‐life with preserved mobility and the ability to talk and eat. However, to ensure a good patient outcome, we need to develop standardized ventilation strategies and protocols for NPV that are based on prospective and controlled studies, which are currently missing in the literature. Also to be determined are the indications and contraindications, such as the specific target populations for which the use of NPV can be most beneficial.

**Reflective questions**

- Why should we consider pressure support ventilation during spontaneous breathing?
- Why would expiratory ventilation assistance be beneficial?
- In which ways can dissipated energy be potentially harmful to the respiratory system?
- What does it mean to implement physiological variability into mechanical ventilation and how could we optimize it?

## CONFLICT OF INTEREST

The authors declare no conflict of interest.

## AUTHORS’ CONTRIBUTIONS

ADSR and GA drafted the manuscript. WH established with ADSR and GA the topics to be included. All authors have read and validated the last version of the manuscript.

## Data Availability

The data used in the current manuscript are available from the corresponding author on reasonable request.
